# Techniques to encourage people to take better care of their eye health

**Published:** 2014

**Authors:** Islay Mactaggart, Paddy Ricard

**Affiliations:** Research Fellow in Disability and Global Health, London School of Hygiene and Tropical Medicine, London, UK. Islay.Mactaggart@Ishtm.ac.uk; Editor, Community Ear and Hearing Health, London, UK.

**Figure:**
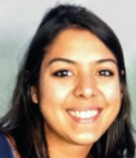
Islay Mactaggart

**Figure:**
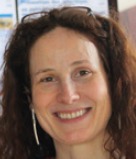
Paddy Ricard

Earlier in this issue, the article ‘How to empower communities to take action on improving eye health’ (page 64) provided the background, rationale and overall approach to empowering communities to improve their eye health. This article provides more information about specific techniques that you might use within your overall strategy, including examples of each technique and when to consider using it.

As mentioned earlier in this issue, it is important that the technique you choose is inclusive, meaning that it is possible for people with different types of disabilities and levels of literacy to benefit. For example, people who are deaf or hearing impaired may need written materials or sign language interpreters. Verbal techniques including plays and puppet shows may be better than written information in illiterate communities. ‘Easy read’^1^ materials (which use short, simple sentences that are clearly presented and supported with helpful images) may help children, people with learning difficulties and those who have lower literacy. Venues should always be accessible for people who use wheelchairs or need support with moving around (e.g. there should be ramps or elevators, not just stairs).

## Information sharing

Information sharing refers to sharing specific messages with the community that can improve their eye health. Depending on the knowledge gaps in the community, the type of information shared may be about what services are available (including where they are, how much they cost, what exact procedures they entail, and what support is available in accessing them) or about specific diseases or infections common in the community (and how they can be identified and treated).

As explained in ‘How to empower communities to take action on improving eye health’, there are many reasons why communities may not be aware of the best practices to improve their eye health, and may not have accurate information about how they can do this and what services are available to support them. Ensuring that the community are aware of these key messages is extremely important.

### How can information be shared?

There are a number of different ways that information can be shared, using different channels of communications and different techniques.

**Figure:**
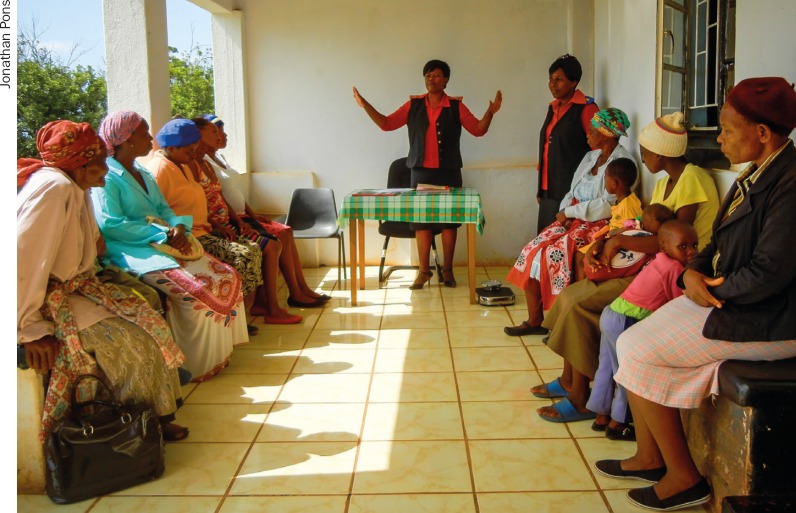
A nurse explains the clinic procedure to the waiting patients and then gives a short talk about a public health topic, such as multiple drug-resistant TB. Before she started to speak, everyone sang a hymn together. Singing is a strong tradition in many African countries and builds a sense of community and connection between the patients and the nurse – all of which supports communication. SWAZILAND

1. **Oral presentations explaining where/ what services are available or how communities can improve their eye health.**
  For example, giving a talk at a church congregation or village meeting on the availability of new cataract services, or giving a health talk in a hospital waiting room (see article on page 76). This is a simple and straightforward approach to communicating information to group gatherings.
2. **Distribution of printed information**
  This is relevant if your audience is literate, or if you are sure that families can find a literate person to read to them. A leaflet that summarises important information about glaucoma treatment, for example, will give opportunities for follow-up discussion and provide contact details if further information or clarification is needed.
3. **Using mass media (print, radio or TV)**
  This is useful to reach a large number of people, as long as you use a medium that is relevant to the community (e.g. newsprint is not appropriate if the majority of the community is not literate). Regular radio or TV messages may be most appropriate to encourage particular strategies (e.g. for trachoma) or to repeat key messages about upcoming screening services. You should make sure you message is broadcast at a time when your target audience is likely to be listening or watching. You could also consider participating in a phone-in radio show where local listeners can ask questions.
4. **Giving a performance that delivers a health message**
  Performance techniques such as street theatre, puppetry or showing a short video can be designed in which the story line, and the characters involved, tell a story about improving eye health. This can make the message more easier to relate to than if the message were delivered on its own. Performance techniques can also provide space for question and answer sessions to ensure that the community has the opportunity to query key points in a relaxed setting.
5. **SMS (text message) reminders**
  SMS reminders sent at timely intervals will reach all members of the community with a cellphone (mobile phone), which is helpful as so many people have cellphones. This can be particularly useful to remind the community when particular services/consultations are available nearby.

## Participatory approaches

A participatory approach is one that allows the community to participate in activities that encourage good eye health behaviour and to actively decide how they will improve their eye health. It aims to foster a sense of shared ownership, which can be more effective than a one-way delivery of information or services (from you to the community).

The community's increased sense of ownership can help to encourage long-term commitment to any behaviour change.

### What type of participatory approaches can be used?

1. Demonstrating the effects and symptoms of a particular health condition Demonstrating to a group of community members the effects and symptoms of an eye condition such as glaucoma (i.e., using custom spectacles to demonstrate tunnel vision), will help them to spot symptoms in themselves and others. If they can experience the effects of an eye disease, they may also have an increased appreciation of the importance of seeking eye health services before an irreversible loss of vision occurs.
2. **Demonstrating a health behaviour.**
  People are more likely to remember something if they have practiced doing it themselves. Going through the steps – of good hand-washing, for example – with a community group will enable you to deal with any questions on the correct way to do it, and it will help to clear up any confusion.
3. **Role-playing a situation. Certain** behavioural changes that you may wish to encourage may cause anxiety or fear in communities. For example, many people can be fearful of operations, e.g. cataract operations, which stops them from seeking treatment. By demonstrating what will happen before, during and after cataract surgery, with a community member playing the role of patient, people can voice their fears about the process in an informal setting when nothing is at stake. This in turn can lead to reduced anxiety about the procedure and better uptake of services.
4. **Involving community members who have already endorsed the intervention/adopted the behaviour/used the service**
  Generally, people trust other members of their community and may relate to them more than strangers who seek to encourage behaviour change. For example, you can ask people who have had successful cataract surgery or successful treatment for glaucoma to talk through their experience, explain the benefits and answer questions from others in the community.
5. **Providing support to community volunteers or community-based workers**
  Community volunteers or paid community workers can provide an ongoing two-way link with the community. Teachers, other local authority staff (eg pre-school workers, elders club staff), and community-based health workers may appreciate training to enable them to assist their local population in improving eye health, for example in organising children or adults' vision screening.

**‘The community's increased sense of ownership can help foster long term commitment to any behaviour change’**

## Facilitating access

Facilitating access means making it easier for people to get the services they need. This is done by addressing the barriers that prevent communities from accessing services, e.g. cost, distance, language, physical barriers (e.g. stairs), and so on.

### What methods can be used to facilitate access?

There are a number of ways in which access to available services can be facilitated:

1. **Distributing materials that can improve eye health.** For example, providing soap for hand-washing, or spectacles to correct refractive error.
2. **Offering free transport to services**
  Organising free group transport to a particular service (such as screening or surgery) lessens the cost burden and can also provide mutual support for community members who may feel less anxious attending services as a group.
3. **Offering free or partially subsidised services.** Subsidising or removing the cost of services can be extremely beneficial in improving eye health when communities do not have much disposable income or have many other competing priorities.
4. **Offering services in the community**
  Organising local screening for cataract or other eye conditions isa very effective way of encouraging good eye health. It is important, however, to make sure that screening activities are linked to the necessary follow-up interventions. This requires a functioning referral network to refer patients on to, as well as coordination and sufficient resources. Alternatively, it may be possible to offer treatment as part of outreach services, e.g. organising cataract surgery at a local clinic when enough patients have been identified.

## Rewarding behaviour change

Rewarding behaviour change means providing incentives to the community to change their eye health behaviour or attend an information session intended to improve their eye health.

A perceived lack of need is often given as a reason why communities do not attend eye services. Rewards may improve uptake. However, you should be aware that this strategy is less sustainable and, if using it is necessary, it may indicate that there remains a lack of clear understanding in the community, or that the service is not really meeting their needs. Consider addressing this before providing incentives.

### What methods can be used to reward behaviour change?

The rewards can be staple foods, soap, etc. or concurrent services that the community feels a stronger need for. You should always avoid providing monetary incentives for people to attend a session or change their behaviour as this is not considered ethical.

## Conclusion

This article has introduced a range of techniques that you can use to encourage people to take better care of their eye health in the community. There is no ‘one size fits all’, and in many cases it may be appropriate to combine more than one technique. Always remember to spend time understanding the community's knowledge gaps and practices before attempting to encourage any changes in behaviour – see ‘How to empower communities to take action on improving eye health’ on page 64.
